# AGK regulates the progression to NASH by affecting mitochondria complex I function

**DOI:** 10.7150/thno.69826

**Published:** 2022-04-04

**Authors:** Nan Ding, Kang Wang, Haojie Jiang, Mina Yang, Lin Zhang, Xuemei Fan, Qiang Zou, Jianxiu Yu, Hui Dong, Shuqun Cheng, Yanyan Xu, Junling Liu

**Affiliations:** 1 Department of Biochemistry and Molecular Cell Biology, Shanghai Key Laboratory for Tumor Microenvironment and Inflammation, Shanghai Jiao Tong University School of Medicine, Shanghai, China.; 2 Shanghai Institute of Immunology, Department of Immunology and Microbiology, Key Laboratory of Cell Differentiation and Apoptosis of Chinese Ministry of Education, Shanghai Jiao Tong University School of Medicine, Shanghai, China.; 3 Department of Hepatic Surgery VI, Eastern Hepatobiliary Surgery Hospital, Second Military Medical University, Shanghai, China.

**Keywords:** mitochondrial respiratory chain, NDUFS2, fatty acid metabolism, mitochondrial ROS

## Abstract

**Background:** Impaired mitochondrial function contributes to non-alcoholic steatohepatitis (NASH). Acylglycerol kinase (AGK) is a subunit of the translocase of the mitochondrial inner membrane 22 (TIM22) protein import complex. AGK mutation is the leading cause of Sengers syndrome, characterized by congenital cataracts, hypertrophic cardiomyopathy, skeletal myopathy, lactic acidosis, and liver dysfunction. The potential roles and mechanisms of AGK in NASH are not yet elucidated.

**Methods:** Hepatic-specific AGK-deficient mice and AGK G126E mutation (AGK kinase activity arrest) mice were on a choline-deficient and high-fat diet (CDAHFD) and a methionine choline-deficient diet (MCD). The mitochondrial function and the molecular mechanisms underlying AGK were investigated in the pathogenesis of NASH.

**Results:** The levels of AGK were significantly downregulated in human NASH liver samples. AGK deficiency led to severe liver damage and lipid accumulation in mice. Aged mice lacking hepatocyte AGK spontaneously developed NASH. AGK G126E mutation did not affect the structure and function of hepatocytes. AGK deficiency, but not AGK G126E mice, aggravated CDAHFD- and MCD-induced NASH symptoms. AGK deficiency-induced liver damage could be attributed to hepatic mitochondrial dysfunction. The mechanism revealed that AGK interacts with mitochondrial respiratory chain complex I subunits, NDUFS2 and NDUFA10, and regulates mitochondrial fatty acid metabolism. Moreover, the AGK DGK domain might directly interact with NDUFS2 and NDUFA10 to maintain the hepatic mitochondrial respiratory chain complex I function.

**Conclusions:** The current study revealed the critical roles of AGK in NASH. AGK interacts with mitochondrial respiratory chain complex I to maintain mitochondrial integrity via the kinase-independent pathway.

## Introduction

Nonalcoholic steatohepatitis (NASH) is associated with inflammation and fibrosis and is the major cause of chronic liver diseases, such as cirrhosis and hepatic carcinoma (HCC) 1, 2. Mitochondrial dysfunction plays an essential role in the progress of hepatic disorders. In NASH, the excessive storage of fats in hepatocytes promotes mitochondrial β-oxidation rate and reactive oxygen species (ROS) production 3. Excessive accumulation of ROS in hepatocytes affects the mitochondrial respiration chain complex and ATP levels, thus causing abnormalities in the mitochondria function 4.

A significantly lower activity of the mitochondrial complex in the liver tissues of NASH patients leads to severe mitochondrial defects and liver dysfunction 5. Mitochondrial respiratory chain complex I (NADH-ubiquinone oxidoreductase) is the primary entry point of electrons to enter the electron transport chain 6. Complex I catalyzes the electron transfer from NADH to ubiquinone and effectuates oxidative phosphorylation 7. Complex I dysfunction is also a major source of ROS accumulation in liver mitochondria, resulting in mitochondrial defect and hepatocyte injury in fatty liver 7.

Acylglycerol kinase (AGK) is a mitochondrial inner membrane protein that participates in the synthesis of mitochondrial inner membrane-specific cardiolipin 8. It phosphorylates monoacylglycerol (MOG) and diacylglycerol (DAG) to form lysophosphatidic acid (LPA) and phosphatidic acid (PA). Also, AGK is a component of the translocase of the mitochondrial inner membrane 22 (TIM22) complex and facilitates protein anchoring to the mitochondrial inner membrane in a kinase-independent manner 9, 10. AGK mutation causes Sengers syndrome; one clinical case reported a patient with homozygous pathogenic variant c.979A>T; p.K327* exhibiting synthetic liver dysfunction 11. Clinical studies demonstrated a deficiency of the mitochondrial respiratory chain complex I activity in patients with Sengers syndrome, indicating an underlying link between AGK and complex I 12. However, the role of AGK in liver function is yet unknown.

In the present study, we used NASH patient samples, liver-specific CDAHFD- and MCD-induced AGK-deficient mouse, and biochemical technology to elucidate the roles of AGK in NASH progression and the putative mechanisms.

## Materials and methods

### Patients

A total of 18 NASH patients' (10 males and 8 females) liver tissues were collected from the Eastern Hepatobiliary Surgery Hospital (Shanghai, China) according to the guidelines between 2010 and 2019 13. Blood routine parameters, including white blood cells, red blood cells, and biochemical markers [total cholesterol, high-density lipoprotein (HDL), low-density lipoprotein (LDL), alanine aminotransferase (ALT), and aspartate aminotransferase (AST)] were showed in the table (Supplemental Table 1). Four control liver tissue sections were collected from normal tissue adjacent to hepatocellular carcinoma by liver cancer resection. Written informed content was obtained from all subjects prior to participation in the study. The tissue samples of NASH were diagnosed by a pathologist and collected according to an established protocol approved by the Ethics Committee of Eastern Hepatobiliary Surgery Hospital. The immunohistochemistry (IHC) staining of AGK in liver sections used rabbit-anti human AGK antibody (Invitrogen, Carlsbad, CA, USA). The staining intensity was analyzed using Image Pro Plus 6.0 analysis software (Media Cybernetics, Rockville, MD, USA).

### Mice

To obtain liver-specific AGK-deficient mice (*Agk^-/-^*), *Agk^f/f^* mice with loxP sites flanking the AGK exon 3 (Shanghai Biomodel Organism Science & Technology Development Co., Ltd (NM-CKO-00026, B6.129), Shanghai, China) on C57BL/6 genetic background 14 were bred with *Albumin-Cre* mice (B6.Cg-Tg(Alb-cre)21Mgn/J; The Jackson Laboratory, Shanghai, China) on 129SV/EV genetic background. Then, *Agk^-/-^* mice were backcrossed 9× onto the C57BL/6 background. AGK is a lipid kinase consisting of a highly conserved diacylglycerol kinase (DGK) domain. The G126E mutation eliminates the kinase activity of AGK by abolishing the three glycine loops through which AGK binds to ATP 8. The glycine was changed to glutamic acid in AGK exon 6 in *Agk^G126E/G126E^* knock-in mice (point-mutant, *PM*) (Bioray Laboratories, Inc., Shanghai, China) onto C57BL/6 genetic background by homologous recombination using CRISPR-Cas9 method 15.

Choline-deficient high-fat diet (CDAHFD) is a method of replicating human NASH phenotypes. Eliminating choline results in impaired lipid export from the liver, causing the progression of NASH 16. Methionine choline-deficient diet (MCD) is another most widely used diet to induce NASH. MCD is a reproducible model, consistently inducing a phenotype of severe NASH 17. In the NASH model, *Agk^-/-^* mice and *Agk^f/f^* mice (6-8-weeks-old), weighing 20-25g, were randomly divided into four groups: *Agk^-/-^* mice treated with CDAHFD or MCD, *Agk^f/f^* mice with CDAHFD or MCD, *Agk^-/-^* mice with chow diet, and *Agk^f/f^* mice with chow diet. The mice were fed CDAHFD (45 kcal% fat; Research Diets, New Brunswick, NJ, USA), MCD (Research Diets), and standard diet for 2 weeks each, respectively 18. The final induction status was determined by the degree of liver damage. *PM* and wild-type (*WT*) mice were fed the same diet. The body weights of mice were monitored and recorded periodically. Additionally, animals were maintained in temperature-controlled specific pathogen-free (SPF) rooms with fixed 12-h light/dark periods, optimal humidity, and sufficient water and diet. The Shanghai Jiao Tong University School of Medicine Animal Care and Use Committee approved the animal research.

Serum was collected from CDAHFD-, MCD-, and chow diet-treated mice. The serum levels of ALT, AST, triglyceride (TG), and cholesterol (CHOL) were measured using a BECKMAN automatic biochemistry analyzer (BECKMAN, Brea, CA, USA). Hematoxylin-eosin staining (H&E) and Oil red O staining were performed to detect the morphological changes and lipid content. The liver fibrosis was assessed by Sirius red and Masson staining. The liver sections were incubated with antibodies against α-smooth muscle actin (αSMA) (Cell Signaling Technology, Danvers, MA, USA) and CD45 (CST) to reflect hepatic stellate cell activation and inflammatory cell infiltration. Image J analysis software (National Institutes of Health, Bethesda, MD, USA) was used for quantification. Fibrosis and hepatic inflammation genes, such as collagen type I alpha 2 (*Col1α2*), collagen type III alpha 1 (*Col3α1*), transforming growth factor-β1 (*Tgfβ1*), interleukin-6 (*IL-6*), *IL-12β*, chemokine (C-C motif) ligand 2 (*Ccl2*), tumor necrosis factor-alpha (*Tnfα*), and interferon-γ (*IFN-γ*) were detected.

### Measurement of oxygen consumption rate (OCR)

OCR was measured using an XF96 extracellular analyzer (Agilent, Shanghai, China). Hepatocytes were isolated using the collagenase Ⅳ perfusion method and seeded at a density of 2.4×10^5^ cells per Cell-Tak (Corning, NY, USA) coated 96-well plate 19. For OCR measurement, 1 μM oligomycin, 0.5 μM fluoro-carbonyl cyanide phenylhydrazone (FCCP), and 0.5 μM rotenone/antimycin A (ROT/AA) were added sequentially. All the reagents were derived from the Seahorse XF Cell Mito Stress Test Kit (Agilent).

### Immunofluorescence and confocal microscopy

AGK-Flag, NDUFS2-HA, and NDUFA10-HA plasmids were transfected into LO_2_ cells using Lipofectamine 2000 (ThermoFisher, Waltham, MA, USA). The cells were stained with Flag antibodies (Sigma-Aldrich, Shanghai, China), HA antibodies (Proteintech, Rosemont, IL, USA), DAPI (Yeasen, Shanghai, China), and mitochondria-targeting dye (ThermoFisher), respectively. Hepatocytes were isolated from *Agk^-/-^* and *Agk^f/f^* mice, as described previously 19. Cells were stained with AGK (Santa Cruz Biotechnology, Santa Cruz, CA, USA), NDUFS2 (Abcam, Cambridge, UK), NDUFA10 antibodies (Santa Cruz Biotechnology), and Lamp1 (Lysosomal-associated membrane protein 1) antibodies (Abcam), respectively. Immunofluorescence images were obtained using a laser scanning confocal microscope (ZEISS, Oberkochen, Germany) equipped with an Airyscan module on the Shanghai Jiao Tong University School of Medicine platform. LAS X 3.3.3 (Leica, Wetzlar, Germany) software was used for data acquisition and image processing.

### Statistical analysis

All statistical analyses were carried out using GraphPad Prism 8 software (La Jolla, CA, USA). The comparisons between groups were carried out with using a two-tailed paired Student's *t*-test. *p*≤0.05 indicated a statistically significant difference.

## Results

### AGK expression levels are decreased in the livers of NASH patients

**Figure 1:**
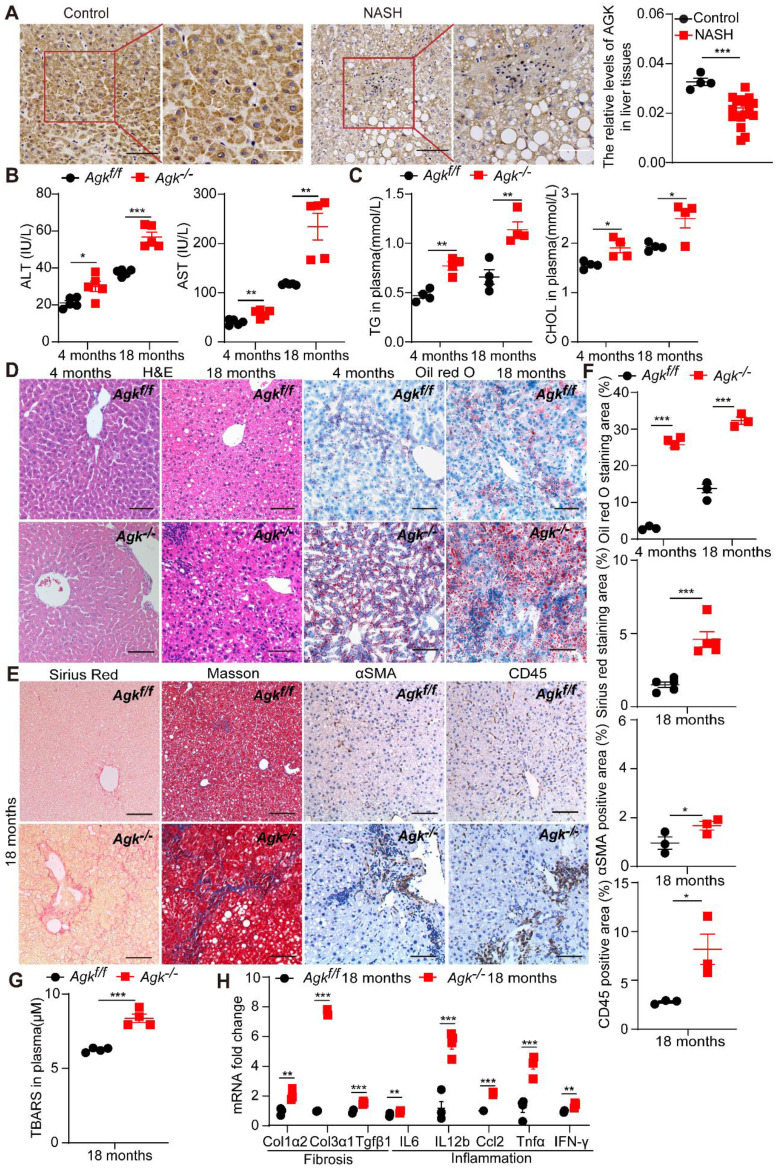
** Liver AGK expression level decreases in NASH patients, and AGK deficiency leads to NASH in mice.** (**A**) Representative liver IHC and positive area analysis of NASH patients (n = 18) and control tissues (n = 4). Black scale bar: 50 µm; White scale bar: 20 µm. ***p < 0.001. (**B-E**) Serum levels (**B-C**), Liver H&E, Oil red O staining (**D**), Sirius red and Masson staining, and immunostaining for αSMA and CD45 (**E**) in *Agk^f/f^* and *Agk^-/-^* mice at the age of 4 months and 18 months (n = 4-5/group; *p < 0.05, **p < 0.01, ***p < 0.001). ALT, alanine aminotransferase; AST, aspartate aminotransferase; TG, triglyceride; CHOL, cholesterol. Black scale bar: 50 µm. (**F**) Quantification of Oil red O, Sirius red, and αSMA, CD45 positive areas (n = 3,5/group; *p < 0.05, **p < 0.01, ***p < 0.001). (**G**) The TBARS levels of plasma in *Agk^f/f^* and *Agk^-/-^* mice at the age of 18 months. TBARS, thiobarbituric acid reactive substances ***p < 0.001. (**H**) mRNAs levels of fibrosis and inflammation marker genes in *Agk^f/f^* and *Agk^-/-^* mice (n = 4/group; **p < 0.01, ***p < 0.001).

To assess the role of hepatic AGK in NASH, IHC analysis was utilized to determine AGK expression in human liver samples from 18 NASH patients. AGK was widely distributed in normal liver tissues, while the levels decreased dramatically in NASH livers, especially in the areas of lymphocytic infiltration. The average AGK expression was significantly lower in NASH liver tissues than in control tissues (Figure 1A). The clinical characteristics and fibrosis scores of the patients are shown in Supplemental Table 1. This phenomenon indicated that AGK is involved in the progression of NASH. Next, the public data repositories were searched for data on array-based mRNA expression of NASH patient livers (GSE48452) 20. Snap-frozen liver biopsies were classified histologically using the nonalcoholic fatty liver activity score. The data analysis from normal controls (n = 12) and NASH patients (n = 14) did not find any difference in the mRNA levels between normal controls and NASH patients (Figure S1A). These results implied that there is no difference in the AGK transcript level. Furthermore, a CDAHFD-induced NASH mouse model was carried out to elucidate the role of AGK in the NASH. Lysosomes are critical for facilitating the degradation of cellular components 21. Lysosomal-associated membrane protein 1 (LAMP1) is a classical lysosome marker 22. The extent of the co-localization of AGK with lysosomes was significantly enhanced in CDAHFD-induced NASH mouse hepatocytes compared to control mouse hepatocytes (Figure S1B). The protein levels of AGK were decreased in CDAHFD-induced NASH mouse hepatocytes (Figure S1C). We also found that the autophagy/lysosome inhibitor chloroquine, but not proteasome inhibitor MG132 restored the protein levels of AGK in NASH mouse hepatocytes (Figure S1C), indicating that the reduction of AGK in NASH underwent a lysosome degradation dependent pathway.

### AGK deficiency causes NASH

Liver-specific AGK-deficient mice (*Agk^f/f^ Alb-Cre^+^*, *Agk^-/-^*) were established to access the role of AGK in the liver. To study the development of liver pathologies, *Agk^-/-^* and *Agk^f/f^* mice were monitored for 18 months in temperature-controlled SPF rooms. At the age of 4 months, the plasma ALT, AST, TG and CHOL levels were elevated in *Agk^-/-^* mice (Figure 1B-C). *Agk^-/-^* mouse livers exhibited mild hepatocyte damage, such as nuclear fracture and lipid droplet accumulation (Figure 1D). Oil red O staining of liver sections showed increased lipid accumulation in *Agk^-/-^* mouse livers (Figure 1D). These findings illustrated that liver AGK deficiency causes mild symptoms of NASH on chow diet-fed mice. Furthermore, at the age of 18 months, ALT, AST, TG, and CHOL levels increased gradually in *Agk^-/-^* mice plasma (Figure 1B-C). Aged mice lacking hepatocyte AGK also caused more hepatocyte ballooning (H&E and Oil red O staining), fibrosis (Sirius red and Masson staining), hepatic stellate cell activation (αSMA staining), and lymphocytic infiltration (CD45 staining) compared to *Agk^f/f^* mice (Figure 1D-F). Plasma thiobarbituric acid reactive substances (TBARS) level indicated that the lipid peroxidation levels are elevated in *Agk^-/-^* mice (Figure 1G). The mRNAs levels of hepatic fibrosis-associated markers, such as *Col1α2*, *Col3α1*, and *Tgfβ1* were significantly enhanced in *Agk^-/-^* hepatocytes (Figure 1H). Similarly, the mRNAs levels of hepatic inflammatory cytokine, such as *IL-6*, *IL-12b*, *Ccl2*, *Tnfα*, and *IFN-γ* were elevated (Figure 1H). These results suggested that AGK deficiency in hepatocytes leads to liver injury, lipid accumulation, and eventually NASH.

### AGK deficiency accelerates CDAHFD-, MCD-induced NASH

**Figure 2:**
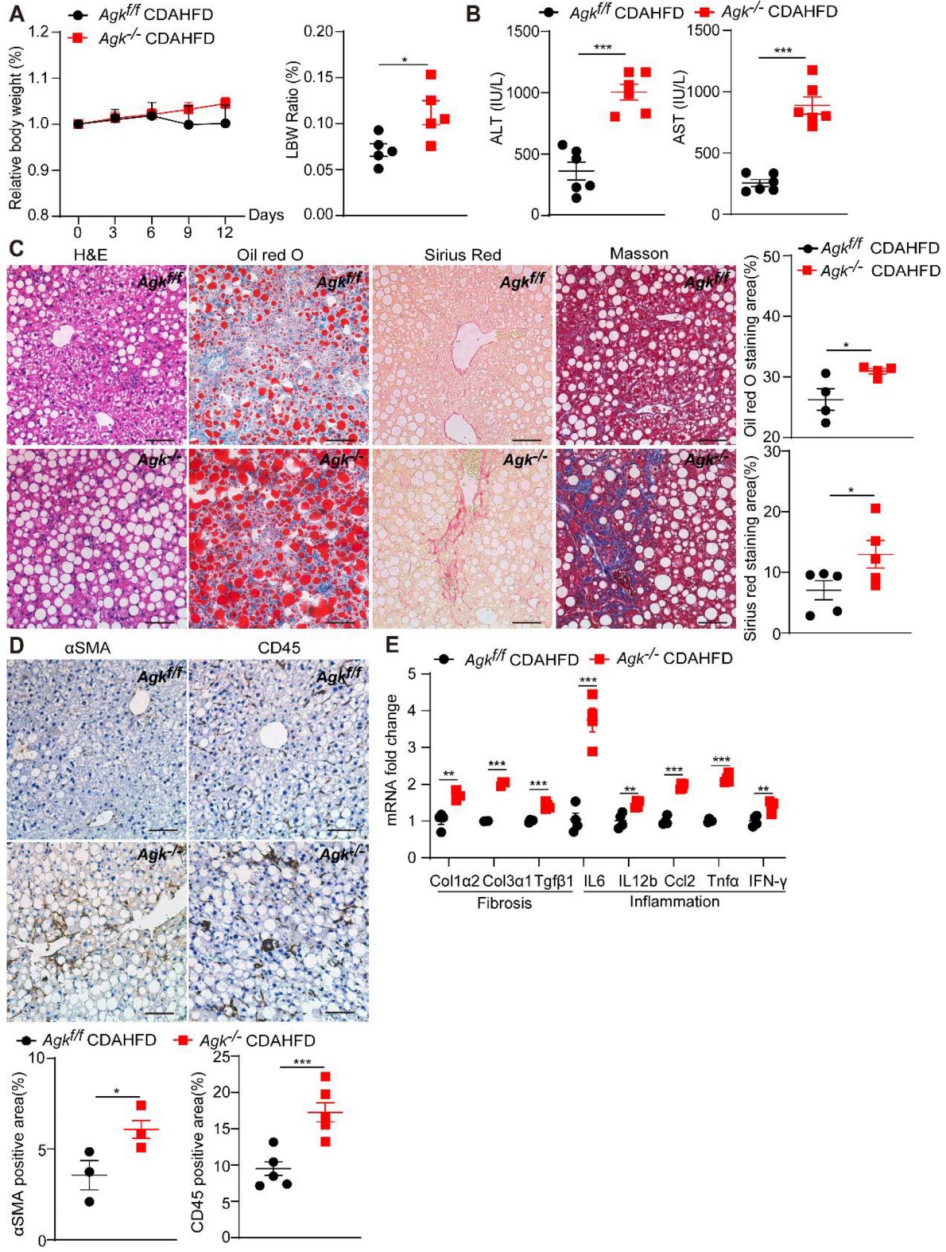
** AGK deficiency promotes NASH.** (**A**) Body weight curves and liver weight to body weight (LBW) ratio of* Agk^f/f^* and *Agk^-/-^* mice in the CDAHFD model (n = 5-6/group; *p < 0.05). (**B-D**) Liver serum levels (**B**), H&E, Oil red O, Sirius red, Masson staining, and quantification of positive areas (**C**), immunostaining for αSMA, CD45, and quantification (**D**) of *Agk^f/f^* and *Agk^-/-^* mice on CDAHFD (n = 3-6; *p < 0.05, ***p < 0.001). Black scale bar: 50 µm. (**E**) mRNAs levels of fibrosis and inflammation marker genes in *Agk^f/f^* and *Agk^-/-^* mice livers in the CDAHFD group (n = 4; *p < 0.05, **p < 0.01, ***p < 0.001).

To further investigate the role of AGK in NASH, *Agk^f/f^* and *Agk^-/-^* mice were fed CDAHFD and control diet for 2 weeks. The bodyweight of AGK-deficient mice was similar to that of the control mice under CDAHFD, but had an increased ratio of liver weight to body weight (Figure 2A). The data also revealed that AGK-deficient mice developed more severe NASH phenotypes than *Agk^f/f^* mice after CDAHFD feeding: higher levels of serum ALT and AST (Figure 2B), more severe lipid accumulation and fibrosis, and greater steatosis (Figure 2C). Despite the significant liver injury, AGK-deficient mice had an increased number of hepatic stellate cells and inflammatory cells compared to *Agk^f/f^* mice (Figure 2D), which was further confirmed by real-time quantitative PCR (qPCR) analysis of hepatic *IL-6*, *IL-12β*, *Ccl2*, *Tnfα*, and *IFN-γ* mRNA levels (Figure 2E). qPCR analysis also showed that AGK-deficient mice had greater hepatic levels of fibrosis-associated markers, including *Col1α2*, *Col3α1* and *Tgfβ1* (Figure 2E). To validate the phenotypes, *Agk^f/f^* and *Agk^f/f^* mice were fed MCD for 2 weeks. The data revealed that AGK-deficient mice developed more severe phenotypes of NASH than control mice after MCD feeding (Figure S2). Therefore, AGK deficiency greatly aggravates the progression of NASH.

### AGK regulates the progression of NASH in a kinase-independent pathway

**Figure 3:**
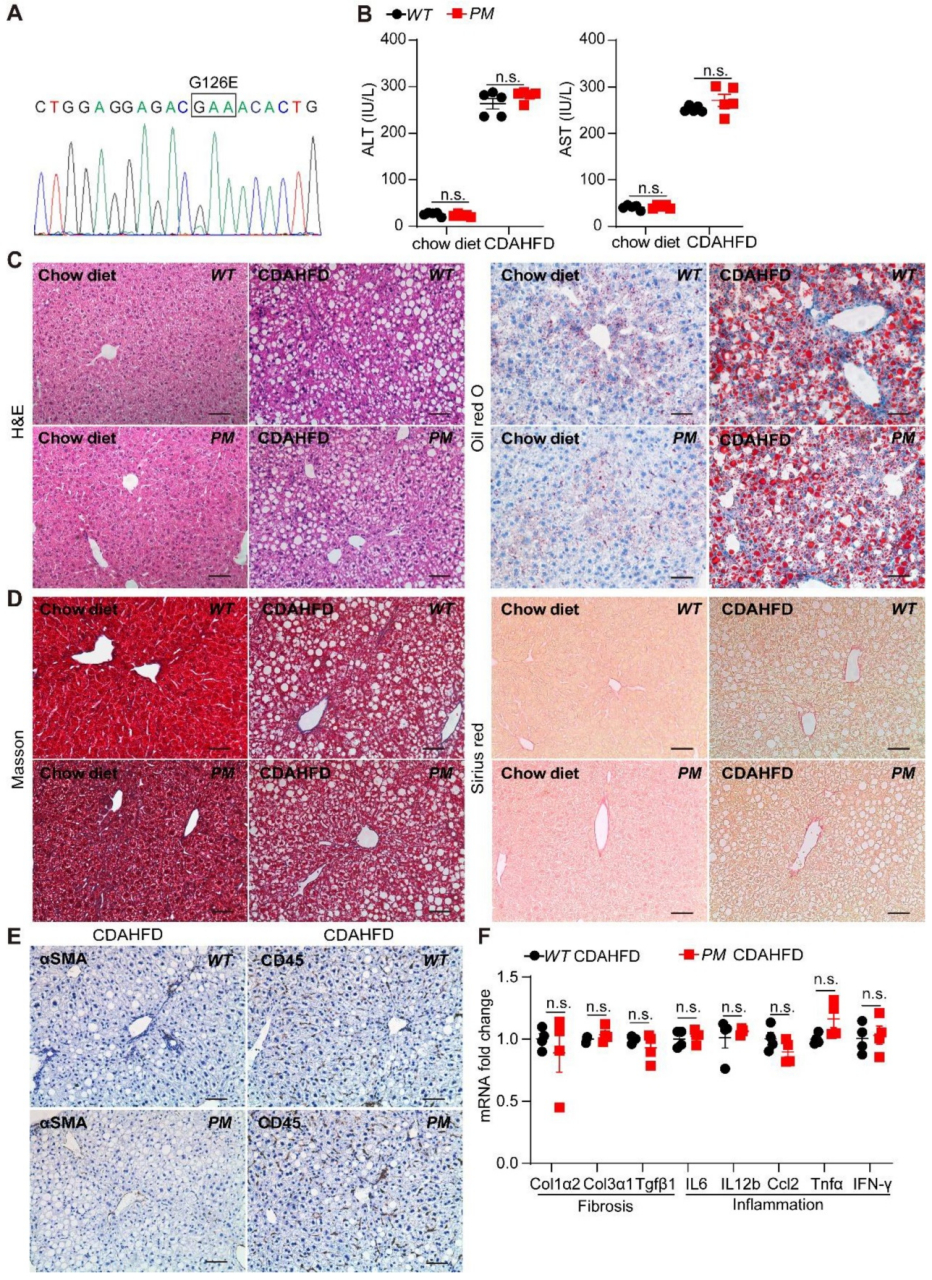
** AGK G126E mutant has no effect on liver function.** (**A**) Genotyping of AGK-G126E *PM* mice by sequencing. (**B-F**) Serum levels (**B**), liver H&E and Oil red O (**C**), Masson and Sirius red staining (**D**), immunostaining for αSMA and CD45 (**E**), and mRNAs levels of fibrosis and inflammation marker genes (**F**) in *PM* and *WT* mice on chow diet and CDAHFD, respectively (n = 4-5/group; n.s., not significant). Black scale bar: 50 µm.

AGK is a lipid kinase that regulates the synthesis of mitochondrial cardiolipin. Reportedly, AGK G126E mutation inhibits AGK kinase activity 15. Furthermore, AGK is also a subunit of the TIM22 transporter complex in the mitochondrial inner membrane. However, the integrity of the TIM22 complex is maintained in the presence of AGK G126E mutation, indicating that AGK plays two different roles in the mitochondria via kinase-dependent and kinase-independent pathways 9, 10. To elucidate the role of AGK kinase activity in livers, AGK G126E point-mutant (*PM*) mice were established and the genotype was confirmed by Sanger sequencing (Figure 3A). The results presented in Figure 3B showed that the plasma ALT and AST levels were similar to *PM* and *WT* mice on chow diets. The results of liver sections staining showed a similar level of lipid accumulation and steatosis in the livers from *PM* and *WT* mice (Figure 3C-D).

Furthermore, *PM* and *WT* mice were fed CDAHFD for 2 weeks. AGK kinase defect did not affect the plasma ALT and AST levels under CDAHFD (Figure 3B). No difference was detected in lipid accumulation, fibrosis, and inflammation in *PM* and *WT* mice under CDAHFD (Figure 3C-E). AGK G126E mutation did not enhance the mRNAs levels of fibrosis markers, such as *Col1α2*, *Col3α1*, and *Tgfβ1* in hepatocytes (Figure 3F). Moreover, AGK kinase defect had no effect on the infiltration of lymphocytes and the production of inflammatory cytokines of* IL-6*, *IL-12β*, *Ccl2*, *Tnfα*, and *IFN-γ* (Figure 3F). In the MCD-induced mice, no difference was observed between* AGK-G126E PM* mice and *WT* mice on these indexes (Figure S3). Additionally, AGK point mutation did not affect the synthesis of cardiolipin (Figure S4A). These results suggested that the kinase function of AGK did not contribute to lipid accumulation-induced liver damage and steatosis.

### AGK deficiency causes hepatic mitochondrial dysfunction

**Figure 4:**
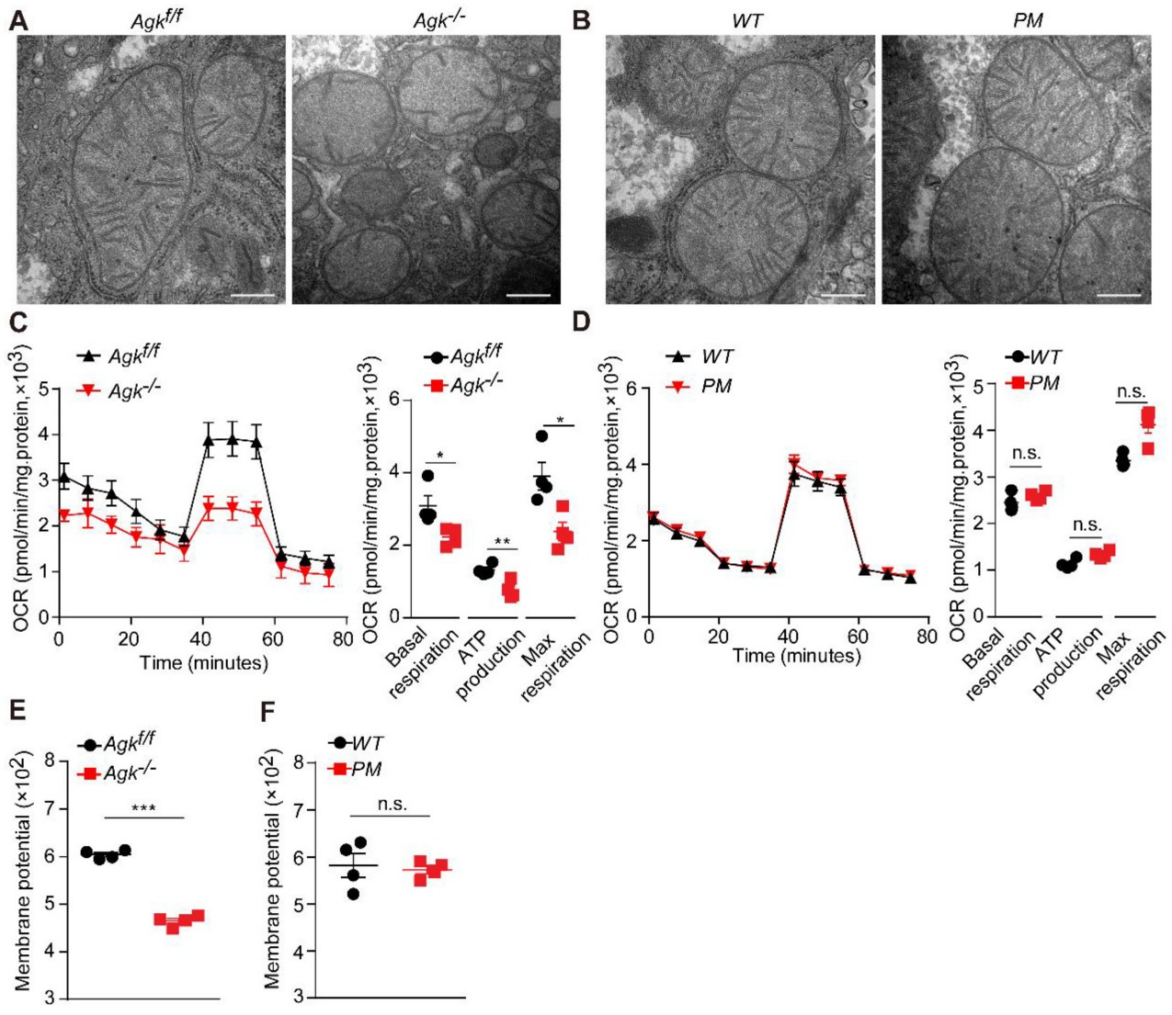
** AGK regulates NASH by affecting mitochondrial function.** (**A-B**) Electron microscopy of mice liver mitochondria*.* **A**,* Agk^f/f^* and *Agk^-/-^* mice; **B**, *PM* and *WT* mice. The bars represent 200 nm. (**C-D**) Extracellular flux analysis of the OCRs of mouse hepatocytes. **C**, *Agk^f/f^* and *Agk^-/-^* mice; **D**,* PM* and *WT* mice. OCR was normalized to the protein amount (n = 4; *p < 0.05, **p < 0.01, n.s., not significant). (**E-F**) The membrane potential of mouse liver mitochondria. **E**, *Agk^f/f^* and *Agk^-/-^* mice; **F**, *PM* and *WT* mice (n = 4; ***p < 0.001, n.s., not significant).

Mitochondria are vital cellular organelles that produce a large amount of ATP via oxidative phosphorylation in the mitochondrial respiratory chain 23. Mitochondrial destruction often aggravates NASH 24, and NASH patients exhibit characteristic mitochondrial abnormalities in hepatocytes, such as loss of mitochondrial cristae and swelling 24. In order to elucidate the effects of AGK deficiency on hepatic mitochondrial morphology, *Agk^-/-^* and *Agk^f/f^* mouse livers were isolated for transmission electron microscopy. *Agk^f/f^* mitochondria had intact cristae, but those of *Agk^-/-^* hepatocytes were significantly decreased and disintegrated (Figure 4A). On the other hand, the mitochondrial cristae remained intact in *PM* hepatocytes (Figure 4B). Furthermore, hepatic AGK deficiency significantly inhibited basic hepatocellular respiration, ATP production, and maximum respiration, as measured by OCR (Figure 4C). Thus, AGK kinase may regulate hepatocellular oxygen consumption since no differences were detected in the respiration between *PM* and *WT* hepatocytes (Figure 4D).

Normal membrane potential is essential for maintaining mitochondrial oxidative phosphorylation and ATP synthesis 25. AGK-deficient hepatocytes exhibited a lower mitochondrial membrane potential than control groups (Figure 4E), but the AGK kinase defect did not affect the membrane potential (Figure 4F). These findings indicated that AGK is crucial for maintaining mitochondrial integrity and function. Also, it is unlikely that AGK kinase activity regulated hepatic mitochondrial function.

### AGK interacts with mitochondrial complex I by anchoring NDUFS2 and NDUFA10 subunits

**Figure 5:**
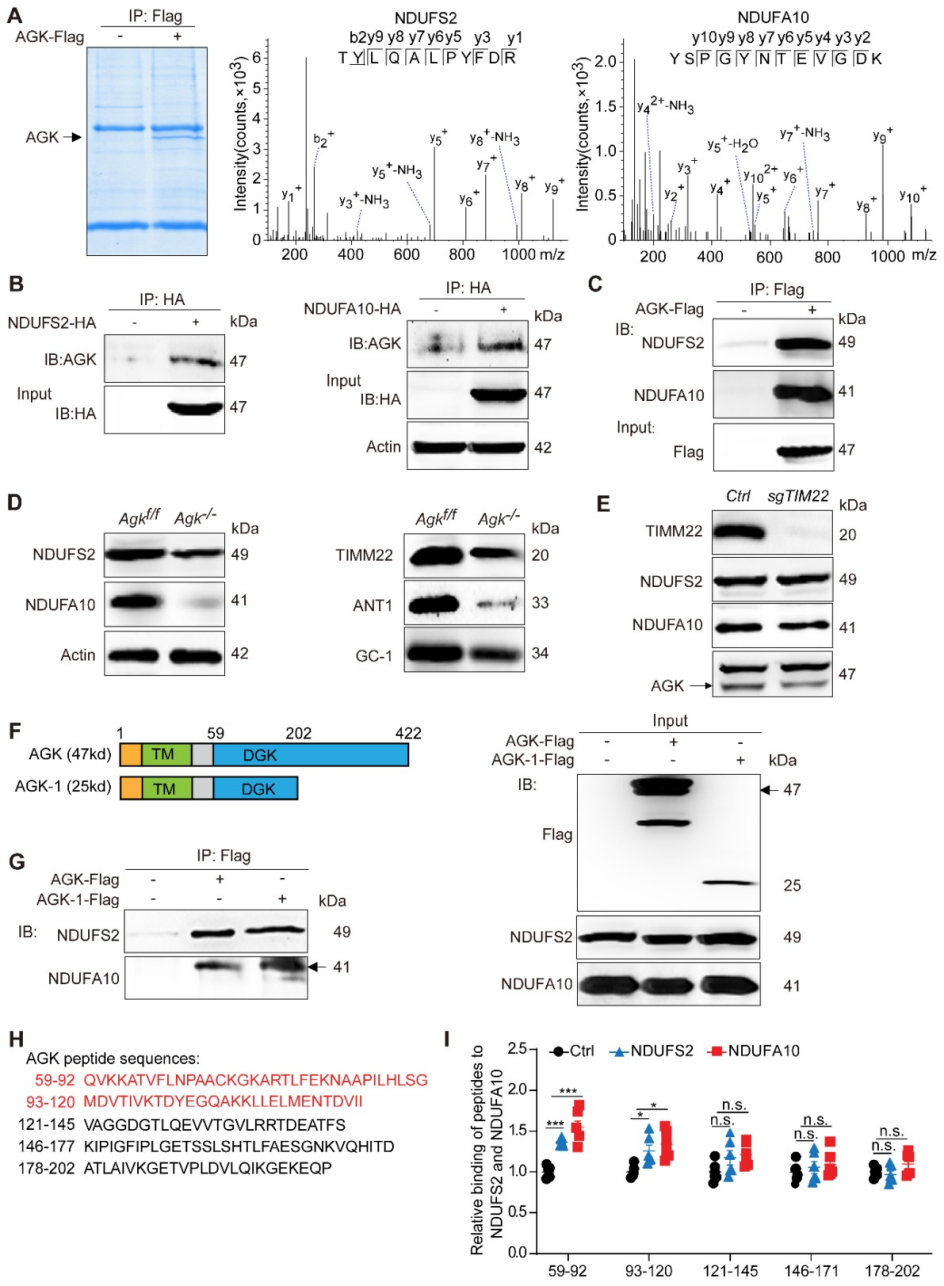
** AGK interacts with mitochondrial complex I by anchoring NDUFS2 and NDUFA10 subunits.** (**A**) Coomassie staining of HEK293T cells transfected with AGK-Flag and vector, and the MS/MS spectrum of NDUFS2 (TYLQALPYFDR) and NDUFA10 (YSPGYNTEVGDK). (**B**-**C**) Coimmunoprecipitation of the lysates of HEK293T cells transfected with NDUFS2-HA, NDUFA10-HA, and AGK-Flag, respectively. (**D**) The expression of NDUFS2, NDUFA10, TIMM22, ANT1 and GC-1 in *Agk^f/f^* mice and *Agk^-/-^* mice hepatocytes. (**E**) The expression of TIMM22, NDUFS2, NDUFA10, and AGK in *sgTIM22* cell line. (**F-G**) Co-immunoprecipitation of the lysates of HEK293T cells transfected with the truncated form of AGK (AGK-1-Flag, 25kd) and full-length AGK (AGK-Flag, 47kd). TM, transmembrane; DGK, diacylglycerol kinase. (**H**) Sequences of five AGK peptides. (**I**) The binding levels of AGK peptides with NDUFS2 and NDUFA10. AGK peptide 1 (59Q-92G), AGK peptide 2 (93M-120I), AGK peptide 3 (121V-145S), AGK peptide 4 (146K-177D) and AGK peptide 5 (178A-202P) (n = 6, *p < 0.05, ***p < 0.001; n.s., not significant).

Mitochondrial respiration complex I is an L-shaped molecule consisting of a hydrophilic arm in the matrix and a hydrophobic arm in the inner membrane 26. As the largest and the most complicated enzyme complex of the oxidative phosphorylation (OXPHOS) system, the structural integrity of mitochondrial complex I guarantees its functional stability 26. Mitochondrial complex I defect also exists in some patients with Sengers syndrome 12. NDUFS2 is a core subunit of the mitochondrial respiration complex I, located in the Q-module 27, while NDUFA10 is located in the hydrophobic protein ND2-module of complex I and involved in the assembling complex I 28. Protein mass spectrometry showed that AGK interacts with the two subunits of mitochondrial complex I, NDUFS2 and NDUFA10 (Figure 5A). Co-immunoprecipitation confirmed that NDUFS2 and NDUFA10 were putative targets of AGK in the regulation of mitochondrial function (Figure 5B-C). Compared to *Agk^f/f^* mice, NDUFS2 and NDUFA10 protein levels were significantly reduced in the liver mitochondria of *Agk^-/-^* mice (Figure 5D), indicating that AGK is essential for maintaining the intensity of mitochondrial complex I.

Reportedly, AGK is a subunit of the TIM22 complex. In the event of AGK deficiency, the carrier proteins ANT1, ANT3, and GC-1 fail to localize on the mitochondrial inner membrane 9, 10. Therefore, a *Timm22*-knockout HEK293T cell line was established to determine whether the AGK/TIM22 complex regulated the respiratory chain complex I function. In AGK-deficient mice, the expression of TIM22, ANT1, and GC-1 was decreased (Figure 5D), which is consistent with a previous report 10. TIM22 deficiency did not affect NDUFS2, NDUFA10, and AGK expression (Figure 5E). These findings indicated that TIM22 complex does not regulate AGK-related mitochondrial complex I function.

AGK is a mitochondrial inner membrane kinase, containing TM and DGK domains 29. To further elaborate the AGK domain that interacts with NDUFS2 and NDUFA10, the N-terminus (1-202P) truncated form of AGK was constructed (Figure 5F). Figure 5G shows that NDUFS2 and NDUFA10 were bound to full-length AGK and AGK-1 (1-202P). Because the truncated form of AGK-2 (1-58A) was not expressed accurately in the mitochondria (results not shown), the sequence of the AGK DGK domain (59Q-202P) was divided into five shorter sequences, which were further synthesized into five peptides and labeled with biotin at the N-terminal (Figure 5H). The binding ability of AGK peptide 1 (59Q-92G), AGK peptide 2 (93M-120I), AGK peptide 3 (121V-145S), AGK peptide 4 (146K-177D), and AGK peptide 5 (178A-202P) to NDUFS2 and NDUFA10 were assessed by enzyme-linked immunosorbent assay (ELISA). NDUFS2 and NDUFA10 specifically bound to AGK peptides 1 and 2 (Figure 5I). To investigate the binding mode of AGK with NDUFS2 or NDUFA10, docking simulation studies were carried out. The results presented in Figure S4B showed that NDUFA10 and NDUFS2 could interact with AGK. Consistently, the binding domain was mainly around 59Q-120I of AGK, which was confirmed by peptides ELISA experiment (Figure 5H-I). The binding sites were Lys82, Asp94, and Thr96 residues in AGK. These results suggested that AGK 59Q-120I directly combines with complex I subunits, NDUFS2 and NDUFA10, to modulate complex I activity.

### AGK has an essential role in maintaining the stability of NDUFS2 and NDUFA10

**Figure 6:**
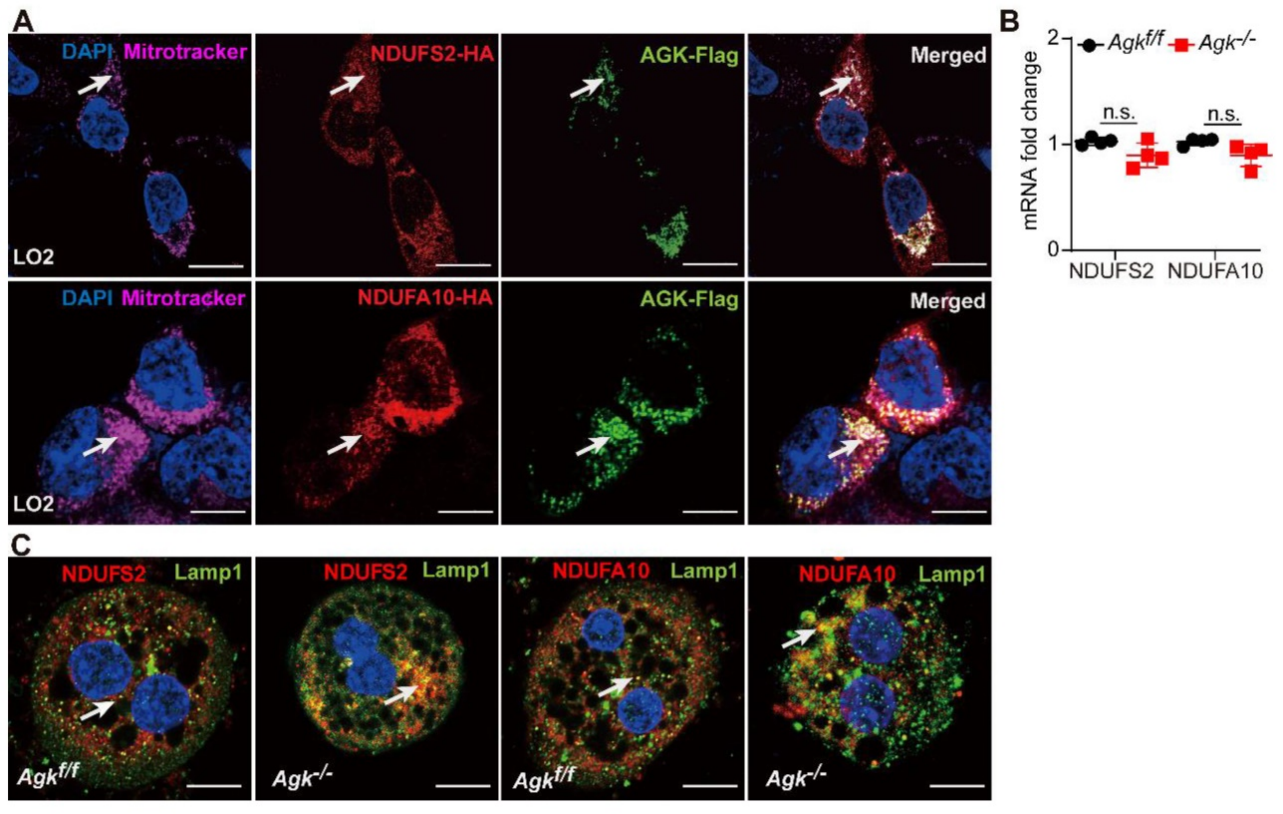
** AGK is important for maintaining the stability of NDUFS2 and NDUFA10.** (**A**) Immunofluorescence images of LO_2_ cell line transfected with NDUFS2-HA, NDUFA10-HA, and AGK-Flag. The cells were stained with Flag antibodies (Alexa Fluor 488), HA antibodies (rhodamine), DAPI, and mitochondria-targeting dye (Mito-Tracker Deep Red, Alexa Fluor 647). The bars represent 5 µm. (**B**) mRNAs levels of NDUFS2 and NDUFA10 in *Agk^f/f^* and *Agk^-/-^* mice (n = 4, n.s., not significant). (**C**) Immunofluorescence images of NDUFS2, NDUFA10 and lysosomes-targeting dye (Lamp1) in the primary hepatocytes of* Agk^f/f^* and *Agk^-/-^* mice. Hepatocytes were stained with NDUFS2 antibodies (rhodamine), NDUFA10 antibodies (rhodamine), DAPI, and Lamp1 (Alexa Fluor 488). The bars represent 10 µm.

AGK was co-localized with NDUFS2 and NDUFA10 in the mitochondria of liver cells, as shown by immunofluorescence staining (Figure 6A). Although, AGK deficiency did not affect the levels of *NDUFS2* and *NDUFA10* mRNAs (Figure 6B), it was associated with the decreased protein levels (Figure 5D), thus confirming that AGK is crucial for maintaining the stability of NDUFS2 and NDUFA10.

Immunofluorescence showed that NDUFS2 and NDUFA10 are co-localized with LAMP1 in *Agk^-/-^* liver cells. The degree of NDUFS2 and NDUFA10 co-localization into lysosomes was significantly enhanced in *Agk^-/-^* hepatocytes compared to *Agk^f/f^* hepatocytes (Figure 6C). Furthermore, compared to the untreated 293T cell line, the protein levels of NDUFS2 and NDUFA10 were decreased in sg*AGK* cells (Figure S4C). We also found that the autophagy/lysosome inhibitor chloroquine, but not proteasome inhibitor MG132 restored the protein levels of NDUFS2 and NDUFA10 in sg*AGK* cells (Figure S4C). These results further confirmed the effect of AGK on NDUFS2 and NDUFA10 degradation via the lysosomal pathway.

Compared to control mice, the levels of NDUFS2 and NDUFA10 proteins were significantly reduced in CDAHFD-induced NASH mice (Figure S4D) but did not affect the levels of *NDUFS2* and *NDUFA10* mRNA (Figure S4D). Immunofluorescence showed that NDUFS2 and NDUFA10 were localized with LAMP1 in CDAHFD-induced NASH mouse liver cells (Figure S4E). The extent of NDUFS2 and NDUFA10 co-localization in lysosomes was significantly enhanced in CDAHFD-induced mouse hepatocytes compared to control mouse hepatocytes (Figure S4E). Furthermore, the expression of NDUFS2 and NDUFA10 was decreased in NASH mice liver cells compared to control mice (Figure S1C). The autophagy/lysosome inhibitor chloroquine, but not proteasome inhibitor MG132 restored the protein levels of NDUFS2 and NDUFA10 in CDAHFD-induced NASH mouse hepatocytes (Figure S1C). Therefore, the reduction of NDUFS2 and NDUFA10 in NASH underwent a lysosome degradation dependent pathway.

### AGK regulates mitochondrial complex I activity and fatty acid accumulation

Mitochondria complex I enzyme activity is the rate-limiting step and regulatory factor of oxidative phosphorylation 7. The decreased activity of complex I blocks the electrons within the respiratory chain. The resulting imbalance between electron input and output stimulates the production of ROS, which induces severe hepatic disease 30. Hepatocyte damage is often accompanied by mitochondrial fatty acid oxidation (FAO) blockage. People with FAO dysfunction have severe hepatic diseases 31. Carnitine is a specific carrier that transports long-chain fatty acids into the mitochondrial inner membrane for β-oxidation. The level of acylcarnitine reflects early FAO balance and mitochondrial stress 32.

**Figure 7:**
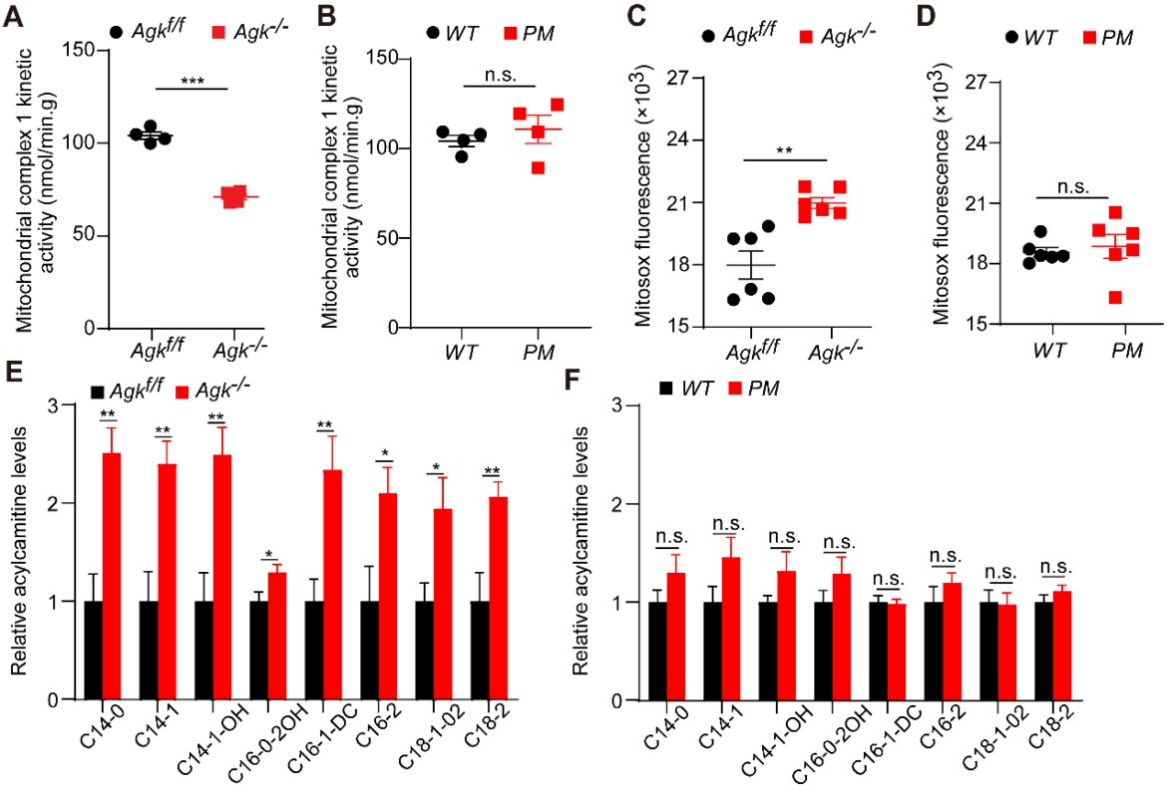
** AGK-deficiency leads to mitochondrial complex I activity defect and fatty acid accumulation.** (**A-B**) The kinetic activity of mitochondrial complex I in mouse hepatocytes. **A**, *Agk^f/f^* and *Agk^-/-^* mice; **B**, *PM* and *WT* mice (n = 4; ***p < 0.001, n.s., not significant). (**C-D**) The ROS levels of mouse liver mitochondria. **C**. *Agk^f/f^* and *Agk^-/-^* mice; **D**. *PM* and *WT* mice (n = 6; **p < 0.01, n.s., not significant). (**E-F**) Analysis of long-chain acylcarnitine species by liquid chromatography-mass spectrometry (LC-MS) in mouse livers. **E**, *Agk^f/f^* and *Agk^-/-^* mice; **F**,* PM* and *WT* mice. (n = 7; *p < 0.05, **p < 0.01, n.s., not significant).

The activity of respiration complex I was significantly decreased in *Agk^-/-^* mice, while no difference was observed in *PM* mice (Figure 7A-B). Mitosox, a fluorescence probe, can be oxidized by mitochondrial superoxide, which reflects the ROS levels in mitochondria 33. Consistently, *Agk^-/-^* mice presented a significant elevation of Mitosox in the liver (Figure 7C). To determine the effects of AGK deficiency on hepatic mitochondrial fat metabolism, the long- and short-chain fatty acids were measured in the livers of *Agk^-/-^* and *Agk^f/f^* mice by liquid chromatography-mass spectrometry. C14, C16, and C18 long-chain fatty acids were significantly accumulated in the cytoplasm of *Agk^-/-^* hepatocytes (Figure 7E), showing that fatty acid metabolism was inhibited in AGK-deficient hepatocytes. When detecting the mRNA levels of genes involved in fatty acid uptake (*CD36*), hepatic lipogenesis (*Srebp* and *Fasn*) and β-oxidation (*Pparg*), *Agk^-/-^* mice showed increased expression of these genes compared to *Agk^f/f^* mice (Figure S4F). The results confirmed that lipid import, lipid production, and fatty acid metabolism in the liver are enhanced in the absence of AGK. Moreover, no significant difference was detected in the livers of *PM* and *WT* mice, suggesting that AGK kinase activity does not participate in the process of hepatic fat metabolism (Figure 7D, F). Together, these phenomena suggested that AGK regulates long-chain fatty acid metabolism, which affects the progression of β-oxidation and mitochondrial functions in an AGK kinase-independent pathway.

Overall, AGK facilitates NDUFS2 and NDUFA10 anchoring on mitochondrial complex I, affecting the function of liver mitochondria and blocking mitochondrial fatty acid metabolism.

## Discussion

NASH is a severe form of NAFLD, characterized by severe hepatocyte injury (ballooning), steatosis, inflammation, and fibrosis 2,34. The pathological progression of NAFLD follows the “three hits”: steatosis, lipotoxicity, and inflammation. Excessive transferred fat in the liver can cause lipotoxicity and organ failure, especially mitochondrial dysfunction. The excessive storage of fat in the liver cells increases the mitochondrial fatty acid β-oxidation rate and ROS production. The imbalance between the excessive accumulation of ROS in liver cells and protective oxidants triggers oxidative stress, which eventually causes liver cell death. Therefore, mitochondrial dysfunction plays a central role in NASH induction 5.

Sengers syndrome is a rare mitochondrial disease caused by AGK mutations and characterized by cataracts, hypertrophic cardiomyopathy, muscle weakness, lactic acidosis after exercise, and liver dysfunction 11. The dual function of AGK as a subunit of the TIM22 complex and as a lipid kinase is impaired mitochondrial protein import and disruption of lipid homeostasis, giving rise to Sengers syndrome 8, 9. Our recent studies showed that AGK modulates the cytoplasmic functions by facilitating PI3K/mTOR signaling in CD8^+^ T cells 15 or JAK2/Stat3 signaling in megakaryocytes 14, indicating that the functional mechanism of AGK is varied among cell types.

In this study, we found that AGK expression was significantly decreased in the liver of NASH patients compared to control tissues, especially in the areas of lymphocytic infiltration. The reduction of AGK in NASH underwent a lysosome degradation dependent pathway. Studies have reported that due to the hepatocellular damage, liver proteins are unstable and tend to be degraded during NASH 35. Particularly, the intracellular lysosomal and proteasome degradation pathways are highly active in the areas of lymphocytic infiltration 21, 35. In this study, we found that AGK levels were dramatically decreased in the livers of NASH through lysosomal but not proteasome degradation pathway. In the damaged hepatocytes, there probably were some modifications in AGK, such as ubiquitination and SUMOylation, made AGK more degradative, especially in the liver cells of lymphocytic infiltration areas. However, this speculation needs more experiments to substantiate.

Furthermore, AGK is mainly located in the mitochondria of hepatocytes, and the kinase deficiency induces mitochondrial crista disappearance, mitochondrial oxidative phosphorylation damage, and fatty acid metabolism disorder in hepatocytes. Therefore, AGK deficiency causes NASH symptoms by interfering with mitochondrial function. However, NASH patients' livers also appear fibrosis and inflammation. Thus, precise methods are required to confirm whether AGK regulates the inflammatory signaling pathways in the liver.

AGK is a multifunctional lipid kinase, which catalyzes the formation of phosphatidic acid, lysophosphatidic acid, and further cardiolipin 8 that participates in mitochondrial phospholipid synthesis, regulating mitochondrial inner membrane permeability and acting as signaling molecules involved in several cell processes 36, 37. Moreover, AGK kinase activity ablation by G126E mutation had no effect on CDAHFD-, MCD-induced NASH, mitochondrial morphology, and mitochondrial function, suggesting that the AGK kinase activity might not participate in NASH. Although, the alterations in CL biosynthesis have been associated with various pathological conditions 38. However, PA can also be synthesized in the endoplasmic reticulum and transported across the intermembrane space (IMS) to the IM 39. Therefore, AGK point mutation did not affect the synthesis of cardiolipin, indicating that AGK may not affect the progression of NASH by affecting the stability of mitochondrial inner membrane-specific cardiolipin.

As the primary entry point of the electron transport chain, mitochondrial respiratory chain complex I is essential for cellular ATP production via the translocation of protons on the inner mitochondrial membrane 26. NASH patients always display complex I dysfunction 5. Complex I is composed of 45 subunits and 15 assembly factors 26. The deficiency of encoding genes and assembly factors leads to complex I deficiency, resulting in mitochondrial disease 40. NDUFS2 and NDUFA10, located at the transmembrane arm of complex I, are crucial for complex I assembly 26. The current study showed that AGK deficiency facilitated the translocation of mitochondrial respiratory chain complex I subunits, NDUFS2 and NDUFA10, to lysosomes and decreased the protein levels, therefore causing the deficiency of mitochondrial respiratory chain complex I. Therefore, the reduction of NDUFS2 and NDUFA10 in NASH underwent a lysosome degradation dependent pathway.

TIM22 complex is a translocase that mediates the import and insertion of multi-pass transmembrane proteins on the inner mitochondrial membrane. Recently, AGK was proved as a subunit of the TIM22 complex that imports mitochondrial carrier proteins 9, 10. AGK deficiency destabilized the TIM22 complex and induced defects in the biogenesis of carrier substrates, causing mitochondrial metabolism disorders 10. Although AGK-deficient mitochondria showed reduced levels of TIM22, TIM22 deficiency had no effects on the expression levels of AGK, NDUFS2 and NDUFA10, suggesting that AGK is essential for regulating the complex I assembly independent of the TIM22 complex.

Further results showed that NDUFS2 and NDUFA10 are associated with AGK transmembrane domain (TMD) and DGK (1-212P). However, short truncated forms of AGK, such as AGK-2 (1-58A), AGK-3 (1-92G and 121V-202P), AGK-4 (1-58A and 93M-202P) and AGK-5 (1-58A and 121V-202P), were not expressed in cells, indicating that transmembrane domain and DGK domain are essential for AGK expression. Reportedly, AGK is anchored to the inner membrane via the N-terminal TM domain 29. Five peptides, with the sequences encompassing AGK (59Q-202P), were synthesized, and the binding assay results suggested that NDUFS2 and NDUFA10 directly bind to the AGK DGK domain (59Q-120I). However, additional precise methods are required to substantiate these findings.

AGK deficiency leads to the blockage of oxidative phosphorylation and accumulation of fatty acids. Furthermore, the mRNA levels of genes, involved in fatty acid uptake, hepatic lipogenesis, and β-oxidation, were increased in *Agk^-/-^* mice compared to *Agk^f/f^* mice. AGK is a lipid kinase localized in mitochondria. However, no report shows that AGK enters the nucleus, indicating that mRNA levels of lipid-related genes cannot be directly regulated by AGK. We speculate that it may be affected by metabolism, such as affecting epigenetics and so on. These mechanisms need to be further explored.

In summary, the current study demonstrated that AGK deficiency triggers the incidence and progress of NASH by blockage of oxidative phosphorylation and accumulation of fatty acids. AGK directly interacts with the mitochondrial complex I subunits, NDUFS2 and NDUFA10, via its DGK domain to affect the mitochondrial functions in the liver. The critical role of AGK in oxidative phosphorylation suggested that the absence of AGK leads to glucose metabolism and amino acid metabolism disorders.

## Supplementary Material

Supplementary methods, figures and table.

## Abbreviations

AGK: acylglycerol kinase
ALT: alanine aminotransferase
αSMA: α-smooth muscle actin
AST: aspartate aminotransferase
Ccl2: chemokine (C-C motif) ligand 2
CDAHFD: choline-deficient and high-fat diet
CHOL: cholesterol
Col1α2: collagen type I alpha 2
Col3α1: collagen type III alpha 1
CQ: chloroquine
DAG: diacylglycerol
DGK: diacylglycerol kinase domain
FAO: fatty acid oxidation
Fasn: fatty acid synthase
FCCP: fluoro-carbonyl cyanide phenylhydrazone
HCC: hepatocellular carcinoma
HDL: high-density lipoprotein
H&E: hematoxylin-eosin staining
IFN-γ: interferon-γ
IHC: immunohistochemistry
IL-6: interleukin-6
LAMP1: lysosomal-associated membrane protein 1
LC-MS: liquid chromatography-mass spectrometry
LDL: high-density lipoprotein
LPA: lysophosphatidic acid
MCD: methionine choline-deficient
MOG: monoacylglycerol
NAFLD: nonalcoholic fatty liver disease
NASH: nonalcoholic steatohepatitis
OCR: oxygen consumption rate
PA: phosphatidic acid
Pparg: peroxisome proliferator activated receptor γ
ROS: reactive oxygen species
ROT/AA: rotenone/antimycin A
Srebp: sterol-regulatory element binding protein
TBARS: thiobarbituric acid reactive substances
TG: triglyceride
Tgfβ1: transforming growth factor-β1
TIM22: the translocase of the mitochondrial inner membrane 22
TMD: transmembrane domain
TNF-α: tumor necrosis factor-alpha
