# Supplementary material for: AGK regulates the progression to NASH by affecting mitochondria complex I function
Source: Theranostics. 2022 Apr 4;12(7):3237–50. doi: 10.7150/thno.69826 (PMC9065199; doi:10.7150/thno.69826)
Supplement: Supplementary file 1 — Supplementary methods, figures and table. [file thnov12p3237s1.pdf]

AGK regulates the progression to NASH by affecting mitochondria complex I function

Nan Ding[1*], Kang Wang[3*], Haojie Jiang[1], Mina Yang[1], Lin Zhang[1], Xuemei Fan[1], Qiang Zou[2], Jianxiu Yu[1], Hui Dong[3], Shuqun Cheng[3#], Yanyan Xu[1#], Junling Liu[1#]

Methods

Construction of plasmids

A truncated form of AGK at the N-terminus (1-202P) from HEK293T cells (The Cell Bank of the Chinese Academy of Sciences, Shanghai, China) was amplified and cloned into the pcDNA3.1-Flag vector. Flag-tagged human full-length AGK vector was described previously [1]. Hemagglutinin (HA)-tagged human NDUFS2 and NDUFA10 sequences were PCR-amplified from HEK293T cells and cloned into the pXJ40-HA vector.

Construction of sg*TIM22* and sg*AGK* by Crispr-cas9

Small guide RNA (sgRNA) targeting the exon 1 of *Timm22* was cloned into pSpCas9(BB)-2A-GFP (PX458) vector [2]. The recombinant plasmid was transfected into HEK293T cells. The cells were harvested after 48 h and single cell-sorting with green fluorescence protein (GFP) at the Core Facility of Basic Medical Sciences (Shanghai Jiao Tong University School of Medicine, Shanghai, China). TIM22 knockout was detected and confirmed by immunoblotting and DNA sequencing. The sg*AGK* cell line targeting the exon 1 of *Agk* was constructed as described previously.

Cell culture and transfection

sg*AGK* cells and HEK293T cells (The Cell Bank of the Chinese Academy of Sciences, Shanghai, China) were maintained in DMEM (Corning, NY, USA) supplemented with 10% fetal bovine serum (FBS) (Gibco, Waltham, MA, USA) at 37 °C in 5% CO_2. To confirm the effect of AGK on NDUFS2/A10 degradation involving lysosomal pathway, cells were treated with 50 μM CQ (chloroquine, lysosomal inhibitor) (Sigma-Aldrich, Shanghai, China), and 20 μM MG132 (MedChemExpress, NJ, USA) for 12 h， respectively.

LO_2 cells were maintained in 1640 media (Corning). Plasmids were transfected into HEK293T cells and LO_2 cells using polyethylenimine linear (PEI), according to the manufacturer's instructions (Yeasen, Shanghai, China). The cells expressing Flag- and HA-tagged fusion proteins were lysed after 48 h. Flag beads (Sigma-Aldrich, Shanghai, China) or HA beads (Santa Cruz Biotechnology, Santa Cruz, CA, USA) were added to enrich the target proteins. Protein samples were separated by polyacrylamide gel electrophoresis.

Cardiolipin detection

Appropriate number of liver tissues (about 30 mg) from *PM* and *WT* mice were weighed and rapidly frozen with liquid nitrogen. These samples were mixed with a homogenizer (Wheaton, IL, USA) by adding ice-cold phosphate-buffered saline (PBS). The supernatants were collected by centrifugation of the above mixtures at 2000 rpm for 20 min. Cardiolipin levels were measured using Cardiolipin Assay Kit

(Yu Bo Biotech Co., Ltd, Shanghai, China) according to the manufacturer's instructions.

Detection of thiobarbituric acid reactive substances (TBARS)

In order to detect the lipid peroxidation levels, fresh blood samples were collected from aged *Agk*$^{-/-}$ and *Agk*$^{f/f}$ mice. The supernatants for the plasma test were collected by centrifugation at 2000 rpm for 10 min. TBARS levels were measured using TBARS Assay Kit (Elabscience, Wuhan, China) according to the manufacturer's instructions.

Mitochondria isolation

Mitochondria were isolated from mice livers using the isolation kit (Sigma-Aldrich). The livers were homogenized and filtered through a 70 μm pore size meshnylon filter. The lysates were further homogenized in the assay buffer with a Dounce tissue grinder (Wheaton). After centrifugation, the mitochondrial fractions were isolated and stored at -80 °C.

Mitochondrial complex 1 kinetic activity detection

Complex I catalyzes the dehydrogenation of NADH to form NAD^+, and the enzyme activity was calculated by measuring the oxidation rate of NADH at 340 nm. The mitochondria were isolated from mice livers. These samples were sonicated by adding 400 μL assay buffer. The mitochondrial complex 1 kinetic activity levels were

measured using the complex 1 kinetic activity assay kit (Solarbio, Beijing, China) according to the manufacturer's instructions.

Mitochondrial membrane potential

Healthy mitochondrial membranes maintain a difference in the electrical potential between the interior and exterior of the organelle, referred to as a membrane potential. Tetramethylrhodamine, methyl ester (TMRM), is a cell-permeant dye that accumulates

in the active mitochondria with intact membrane potentials. Primary hepatocytes of mice were isolated and seeded at a density of 2.4×10^4 cells/well. The absorbance was measured at 570 nm after incubating TMRM (Invitrogen, Carlsbad, CA, USA) at 37 °C for 30 min in the dark. Three replicates were set for each sample.

Mitochondrial reactive oxygen species (ROS) detection

In order to reflect the ROS levels in mouse livers, primary hepatocytes of mice were isolated and seeded at a density of 2.4×10^4 cells/well in a 96-plate well. MitoSOX™ red mitochondrial superoxide indicator (Invitrogen) is a novel fluorogenic dye for the highly selective detection of superoxide in the mitochondria of live cells. The absorbance was measured at 510/580 nm after incubating MitoSOX™ at 37 °C for 30 min according to the manufacturer's instructions.

Enzyme-linked immunosorbnent assay (ELISA)

The overexpressed NDUFS2/A10-HA 293T cell lysates were added to wells coated

with HA antibody, respectively. Then mixtures were incubated with biotinylated AGK peptides at 37 °C for 30 min. The color development catalyzed by horseradish peroxidase was terminated with 2 M sulfuric acid, and the absorption was measured at 450 nm. Protein-protein interaction was calculated by comparing the relative absorbance of the samples with the standards.

Electron microscopy (EM)

Fresh mouse livers were harvested, cut into 1 mm^3 tissue blocks, and fixed in 2.5% glutaraldehyde/phosphate buffer, followed by dehydration with ethanol and acetone and embedding in resin before slicing into ultrathin sections for staining. The platform of Shanghai Jiao Tong University School of Medicine was responsible for the follow-up processing. The 3000× lens electron microscope was used for imaging.

Protein mass spectrometry

pcDNA3.1, AGK-Flag plasmids were transfected into HEK293T cells as the text description. Coomassie blue-stained gel bands were analyzed by mass spectrometry. The mixture of peptides was separated using Zorbax 300SB-C18 peptide trap columns (Agilent Technologies, DE, USA) and tandem mass spectrometry (Thermo Finnigan Q Exactive, MA, USA). Peptides were collected according to the nuclear-mass ratio of 300.00-1800.00. SEQUEST software (Thermo Fisher Scientific, Waltham, MA, USA) was used to search the IPI Human 3.87 database for protein identification. Gel digestion, mass spectrometry analysis, and database search were performed at the

Core Facility of Basic Medical Sciences, Shanghai Jiao Tong University School of Medicine.

Acylcarnitine analysis with liquid chromatography-mass spectrometry (LC-MS)

The middle part of the largest mice liver, about 30 mg, was excised and homogenized in pre-cooled 80% acetonitrile containing internal standard (IS). LC-MS was performed on Agilent 1290 UHPLC system equipped with a binary solvent delivery manager, an auto sampler and column oven, coupled with a 6545 Quadrupole Time of Flight Mass Spectrometer with an electrospray interface (Agilent). The samples were separated by reverse-phase chromatography. The raw data were acquired in full scan positive mode within a mass range of 100-1000 m/z with real-time mass calibration. Each mass response was corrected with the response of the respective IS. Mass Hunter software (Agilent) was used for data processing. Finally, the data were analyzed at the Core Facility of Basic Medical Sciences, Shanghai Jiao Tong University School of Medicine.

Protein-protein docking

The crystal structure of AGK, NDUFS2, and NDUFA10 proteins was downloaded from RCSB Protein Data Bank (http://www.rcsb.org/). The PDB ID of AGK, NDUFS2, and NDUFA10 is 7CGP_B, 5XTD_Q, and 5XTD_w, respectively. The protein-protein docking protocol in MOE1 was applied for molecular docking. The small protein (a small number of residues) is usually set as the ligand and the large

one as the receptor. The multistage method was used for generating and ranking the poses. Starting from a coarse-grained (CG) model to reduce the computational search space, exhaustive sampling is carried out to generate a set of initial poses. Hopf fibration was used to generate a set of uniformly-distributed rotations, and a Fast Fourier Transform (FFT) was used to sample all translations for a given rotation, followed by a minimization process established around a staged convergence protocol. The conformation of the best ranked was selected as the final (probable) binding mode. The docked structures and interface residues were analyzed using the MOE protein contacts module. Molecular graphics were generated using PyMOL.

References

1. Jiang H, Yu Z, Ding N, Yang M, Zhang L, Fan X, et al. The role of AGK in thrombocytopoiesis and possible therapeutic strategies. Blood. 2020; 136: 119-29.

2. Ran FA, Hsu PD, Wright J, Agarwala V, Scott DA, Zhang F. Genome engineering using the CRISPR-Cas9 system. Nat Protoc. 2013; 8: 2281-308.

Figure legends

Figure S1. The expression of AGK in NASH. (A) mRNA levels of *AGK* in healthy (n = 12) and NASH patients (n = 14) livers (n.s., not significant). **(B)** Immunofluorescence images of AGK and lysosomes-targeting dye (Lamp1) in the primary hepatocytes of control mice and CDAHFD-induced NASH mice. Hepatocytes were stained with AGK antibodies (rhodamine), DAPI, and Lamp1

(Alexa Fluor 488). The bars represent 10 μm. (**C**) Expression of AGK, NDUFS2, and NDUFA10 in control mice and CDAHFD-induced NASH mice. CQ, chloroquine.

Figure S2. AGK deficiency promotes MCD-induced NASH. (A-E) Liver serum levels (**A**), mRNAs levels of fibrosis and inflammation marker genes (**B**), H&E and Masson staining (**C**), Oil red O and Sirius red staining (**D**), immunostaining for αSMA and CD45 (**E**) of $Agk^{f/f}$ and $Agk^{-/-}$ mice on MCD (n = 4-5; *$p < 0.05$, **$p < 0.01$, ***$p < 0.001$). Black scale bar: 50 μm.

Figure S3. AGK G126E mutant has no effect on MCD-induced NASH. (A-E) Liver serum levels (**A**), mRNAs levels of fibrosis and inflammation marker genes (**B**), H&E and Masson staining (**C**), Oil red O and Sirius red staining (**D**), immunostaining for αSMA and CD45 (**E**) of *PM* and *WT* mice on MCD (n = 4; n.s., not significant). Black scale bar: 50 μm.

Figure S4. The functions of AGK in NASH. (**A**) Plasma cardiolipin levels of *WT* and *PM* mice (n = 4, n.s., not significant). (**B**) The surface binding model and 3D binding model of AGK and NDUFS2/NDUFA10. AGK is stained slate, NDUFS2 is stained yellow, and NDUFA10 is stained magenta. (**C**) Expression of NDUFS2 and NDUFA10 in 293T and sg*AGK* cell lines. (**D**) Expression of NDUFS2 and NDUFA10 in control and CDAHFD-induced NASH mouse hepatocytes (n = 4, n.s., not significant). (**E**) Immunofluorescence images of NDUFS2, NDUFA10, and Lamp1 in the primary hepatocytes of control and CDAHFD-induced NASH mouse hepatocytes. Hepatocytes were stained with NDUFS2 antibodies (rhodamine), NDUFA10 antibodies (rhodamine), DAPI, and Lamp1 (Alexa Fluor 488). The bars represent 10

μm. (**F**) mRNA levels of genes involved in β-oxidation and lipogenesis in *Agk*$^{f/f}$ and *Agk*$^{-/-}$ mice (n = 4, *$p < 0.05$, ***$p < 0.001$).

Supporting Table 1. Information of NASH patients

Variables	NASH patients (n = 18)
Age (year)	57.5 ± 25.5
Gender-Male (n, %)	10 (56)
Fibrosis score	S0 (n = 4), S1 (n = 9), S2 (n = 2), S3 (n = 1), S4 (n = 2)
ALT (IU/L)	27.2 ± 30.8
AST (IU/L)	28.1 ± 21.9
Total cholesterol (mmol/L)	4.2 ± 1.3
Leukocytes ($\times 10^9$ /L)	5.8 ± 6.6
Erythrocytes ($\times 10^{12}$ /L)	4.4 ± 0.9
HDL (mmol/L)	1.4 ± 0.3
LDL (mmol/L)	2.6 ± 1.1

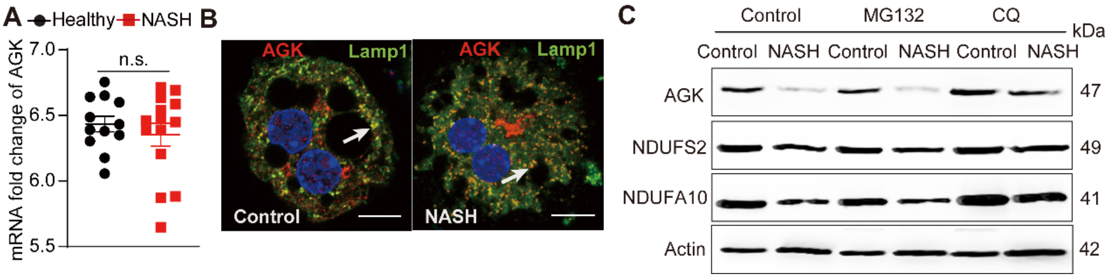

Figure S1

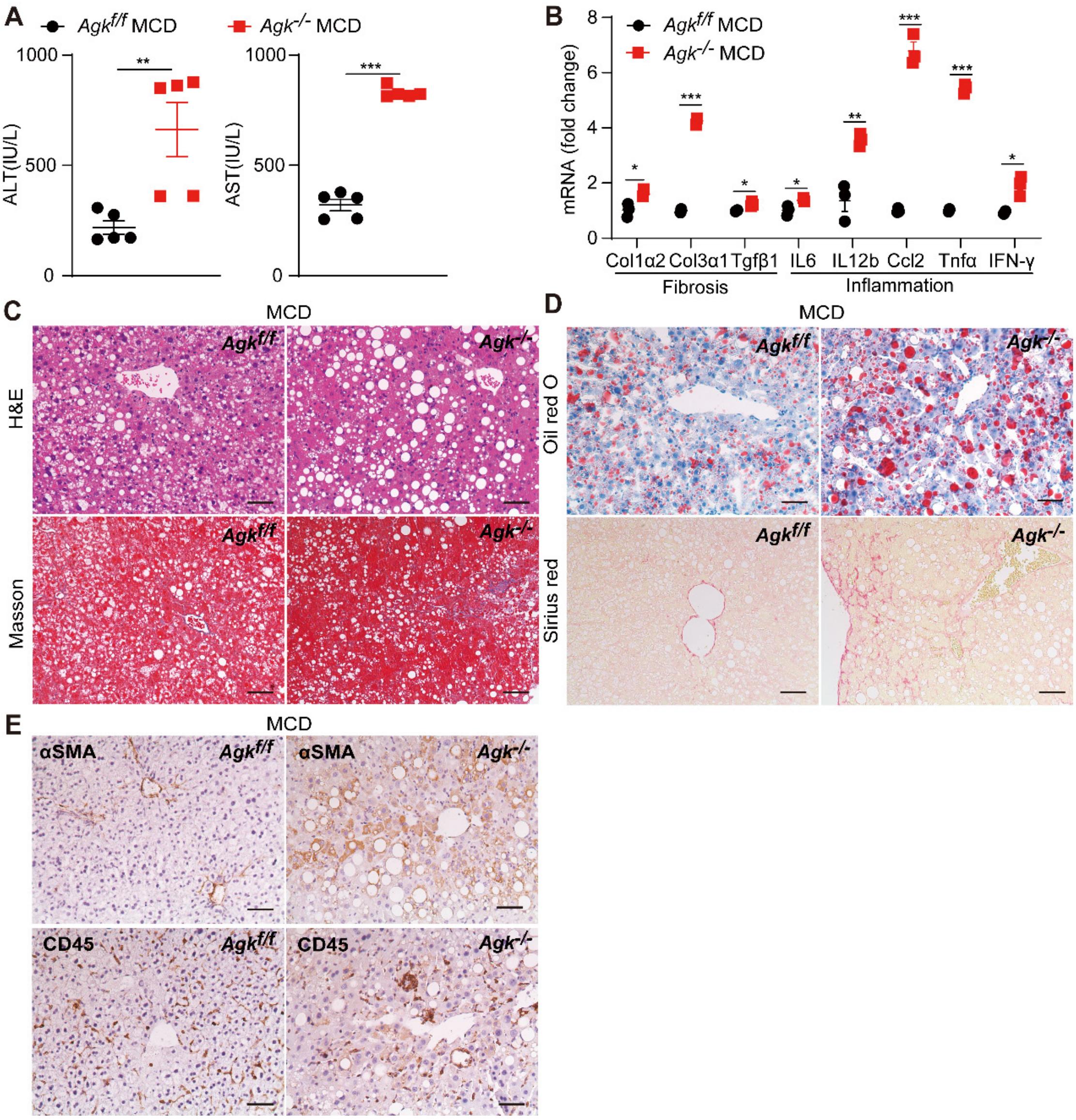

Figure S2

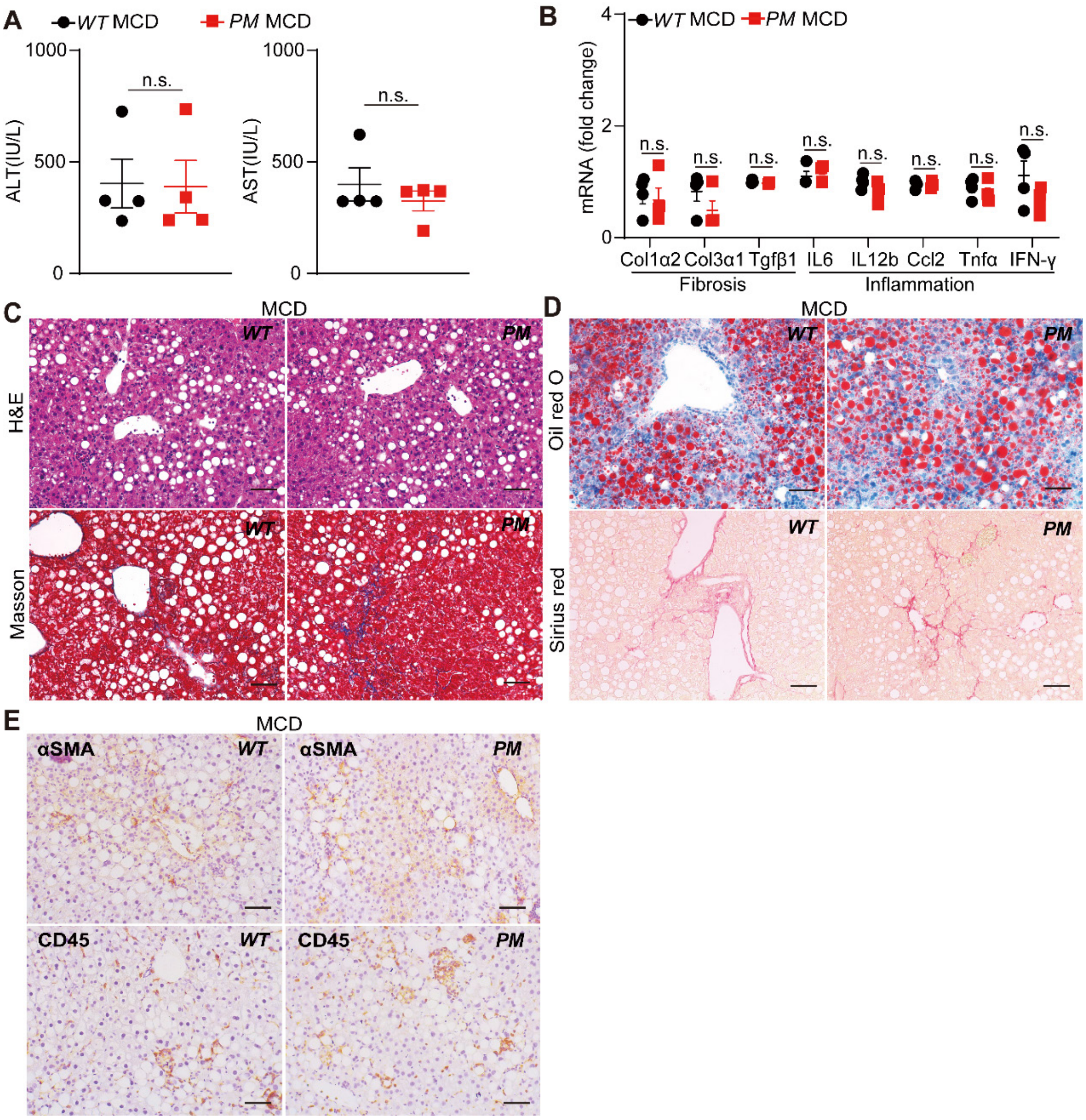
A
WT MCD
PM MCD
1000
500
0
ALT(IU/L)
AST(IU/L)
n.s.
B
4
2
0
mRNA (fold change)
Col1α2 Col3α1 Tgfβ1 IL6 IL12b Ccl2 Tnfα IFN-γ
Fibrosis
Inflammation
C
MCD
H&E
Masson
WT
PM
D
MCD
Oil red O
Sirius red
E
MCD
αSMA
CD45

Figure S3

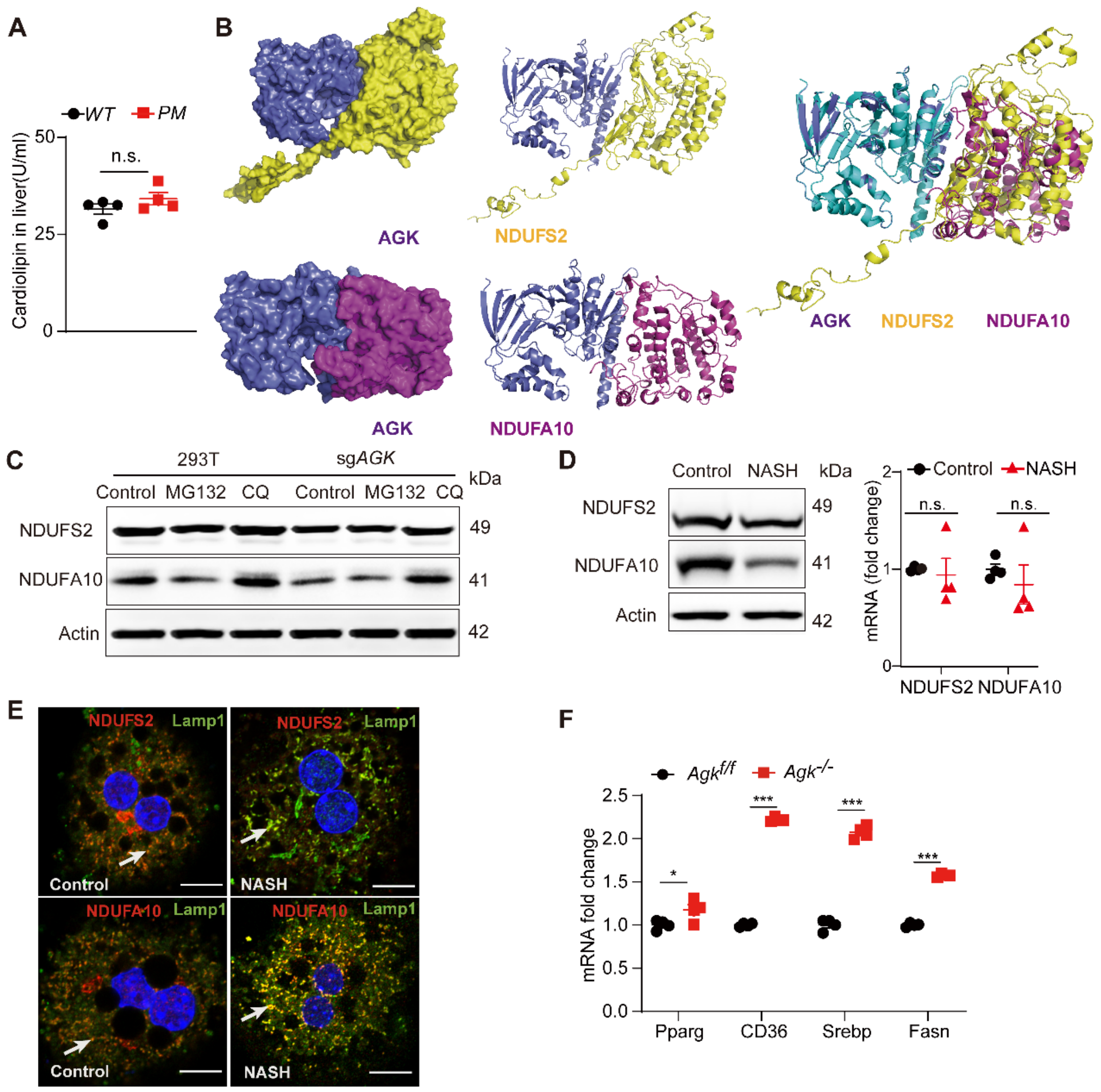
A
B
WT
PM
Cardiolipin in liver(U/ml)
50
25
0
n.s.
AGK
NDUFS2
AGK
NDUFA10
AGK
NDUFS2
NDUFA10
C
293T
sgAGK
Control
MG132
CQ
Control
MG132
CQ
kDa
NDUFS2
NDUFA10
Actin
49
41
42
D
Control
NASH
kDa
NDUFS2
NDUFA10
Actin
49
41
42
Control
NASH
mRNA (fold change)
n.s.
n.s.
NDUFS2
NDUFA10
E
NDUFS2
Lamp1
Control
NASH
NDUFA10
Lamp1
Control
NASH
F
Agkf/f
Agk-/-
mRNA fold change
2.5
2.0
1.5
1.0
0.5
0.0
Pparg
CD36
Srebp
Fasn

Figure S4
